# Morphovolumetric analysis of aneurysm sac and correlation with maximum diameter for post-EVAR surveillance

**DOI:** 10.3389/fcvm.2025.1684466

**Published:** 2025-12-04

**Authors:** Mehmet Ali Türkcü, Emre Külahcıoğlu, Ferit Cetinkaya, Mustafa Dağlı, Hakkı Zafer İşcan

**Affiliations:** 1 TC Saglik Bakanligi Iskenderun Devlet Hastanesi, Iskenderun, Türkiye; 2 Cardiovascular Surgery, TC Saglik Bakanligi Kilis Prof Dr Alaeddin Yavasca Devlet Hastanesi, Kilis, Türkiye; 3 Cardiovascular Surgery, TC Saglik Bakanligi Agri Egitim ve Arastirma Hastanesi, Ağrı, Türkiye; 4 Department of Radiology, TC Saglik Bakanligi Ankara Sehir Hastanesi, Çankaya, Türkiye

**Keywords:** EVAR, volumetric analysis, abdominal aort aneurysm, graft, endovascular

## Abstract

**Introduction:**

Endovascular Aortic Repair (EVAR) is the current treatment of choice for suitable patients undergoing abdominal aortic aneurysm surgery, and post-EVAR follow-up has become an increasingly important topic. This study aims to identify factors influencing the remodeling of aneurysms post-EVAR and assess a more effective follow-up protocol using post-EVAR morphovolumetric analysis.

**Method:**

Between 2019 and 2023, 131 patients who underwent elective Endovascular Aortic Repair (EVAR) at our hospital and patients who had preoperative Computed Tomographic Angiography (CTA) were included in the study. Maximum aneurysm sac diameter, volume, thrombus volume, and infrarenal aortic neck angulation were measured. A post-EVAR volume reduction of 10% or more in the aneurysmal sac was considered a “positive remodeling” based on volumetric analysis. The reliability of post-EVAR surveillance and its correlation with the maximum diameter (Dmax) was evaluated.

**Results:**

Preoperative aneurysm volume larger than 233.5 cm^3^ (*p* = 0.001) and thrombus volume greater than 204 cm^3^ (*p* = 0.002) were associated with secondary interventions. Morphological changes after EVAR included neck dilatation (*p* < 0.001) and decreased neck angulation (*p* < 0.001). An alpha angle >47.5° was associated with Type 1a endoleak (*p* = 0.046). In the follow-up, positive remodeling was observed in 44 out of 131 patients (33.6%). The identification of more than four lumbar arteries preoperatively (*p* = 0.003), the use of Double Antiplatelet Therapy (DAPT) postoperatively (*p* = 0.017), and the presence of any type of endoleak were factors associated with negative remodeling.

**Conclusion:**

Post-EVAR morphological changes include reduced infrarenal aortic lengthening and neck dilatation. Lifelong surveillance is imperative after EVAR. The most effective method of organizing these surveillance protocols may involve a combination of Dmax and volume analysis, allowing for the most economical and reliable monitoring. Dmax is currently the fastest and most reliable data in post-EVAR surveillance. However, following our current study, we observed that relying solely on Dmax can be deceptive and should be correlated with volume analysis data.

## Introduction

Elective infrarenal abdominal aortic aneurysms (iAAA) are treated with Endovascular Aneurysm Repair (EVAR) in approximately 70%–80% of cases (1). This minimally invasive procedure has become the preferred treatment method due to its reduced recovery time and lower procedural risks compared to open surgical repair. However, post-EVAR, about 30% of patients are at risk of requiring secondary interventions within a 10-year period. This significant percentage necessitates close monitoring of EVAR patients who exhibit continuous increments in risk, as early detection of complications can mitigate severe outcomes (2).

Post-EVAR routine imaging has been deemed obligatory to identify complications associated with the graft and mitigate the risk of rupture stemming from the potential expansion of the aneurysmal sac. This close monitoring typically involves assessing the maximum diameter of the aneurysm to detect any undesirable changes. In clinical practice, the most frequently employed measurement method is assessing maximum diameter of aneurysm. This method, while being gold standard, has notable limitations. The measurement of diameter is a two-dimensional and linear assessment, susceptible to influences from the patient's blood pressure, the position during CT scans, and the thickness of CT sections (3). These variables introduce potential inaccuracies, which can mislead the assessment of aneurysm stability.

Some studies suggest that diameter measurements may not accurately reflect changes in the shape of the aorta or alterations in an aneurysm with an amorphous structure (4–6). This is crucial because following EVAR, changes can occur in various regions of the aneurysm due to the radial strength of the endograft and the oversize effect. As such, relying solely on maximum diameter measurement can sometimes yield misleading conclusions, particularly in aneurysms that do not exhibit a change in diameter but may still be experiencing significant morphological changes.

In such cases, volumetric analysis should be considered to complement and support the assessment, providing a more comprehensive understanding of the alterations in the aneurysm. Volumetric analysis offers a three-dimensional perspective, capturing subtle changes that diameter measurements might miss. Keulen et al. have indicated that aneurysm sac volume measurement reflects changes not captured by orthogonal and transverse diameter measurements (7). This method allows for a more nuanced view of the aneurysm, accounting for irregularities and overall volume changes rather than relying on a single maximal dimension.

Recent studies also emphasize the superiority of three-dimensional volumetric analysis over two-dimensional diameter measurements (3, 7, 8). This provides a significant advantage in the accurate monitoring of aneurysms post-EVAR. However, uncertainty persists regarding whether volume measurement will replace diameter measurement in identifying rupture risk and determining clinically significant sac enlargement in abdominal aortic aneurysms (9). The transition to volume measurements presents a shift in clinical practice that requires further validation and acceptance within the medical community.

As a disadvantage, volume measurements take longer time periods than maximum diameter (D max). This increased time requirement, when coupled with potentially higher costs and the need for advanced imaging software, poses a barrier to the routine adoption of volumetric analysis. Nevertheless, the detailed insights it provides could outweigh these drawbacks, leading to better patient outcomes through more precise monitoring and timely intervention.

The objective of our study is to evaluate factors influencing aneurysm remodeling using morphovolumetric measurements. We aim to assess the effectiveness of a follow-up protocol utilizing post-EVAR morphovolumetric analysis for better monitoring. By exploring these advanced measurement techniques, we seek to enhance the accuracy and reliability of post-EVAR surveillance, ultimately improving the long-term prognosis for patients with infrarenal abdominal aortic aneurysms.

## Material and method

Our study was conducted prospectively as an observational single-center study, including 131 patients who underwent elective Endovascular Aneurysm Repair (EVAR) for infrarenal abdominal aortic aneurysms between 2019 and 2023. 175 patients who underwent EVAR were evaluated; of those, 44 patients with missing preoperative or postoperative computed tomography and follow-up data were excluded from the analysis. The study received ethical approval from Ankara City hospital's Institutional Review Board (Approval No: 1428). Informed consent was obtained from all patients. Patients with preoperative and postoperative computed tomographic angiography (CTA) with a slice thickness of 0.625 mm were included. Patients admitted urgently with ruptured aneurysms, as well as those lacking control CTA and graft measurement data, were excluded from the study. All EVAR procedures were performed by the same cardiovascular surgical team.

Data were collected and categorized into preoperative, intraoperative, postoperative, and follow-up periods. Preoperative data comprised patients' demographic information, preoperative CTA diameter measurements, three-dimensional (3D) total aneurysm sac volume measurement, thrombus volume measurement, and thrombus density measurement. Intraoperative data included graft measurements, procedure duration, fluoroscopy, and contrast amounts. Postoperative data encompassed durations of intensive care and hospital stay, complications, follow-up data, CTA diameter measurements, 3D total aneurysm volume, thrombus volume, thrombus density measurement, endoleaks, secondary interventions, and long-term mortality.

Follow-up computed tomographic angiography (CTA) was routinely performed at 1 month, 6 months, 12 months, and annually thereafter following EVAR, in accordance with our institutional protocol. Morphovolumetric and diameter measurements were obtained at each of these timepoints and compared with preoperative baseline values to assess aneurysm sac remodeling over time.

The primary endpoints of our study were to determine the impact of morphological parameters and morphovolumetric measurements on aneurysm remodeling following Endovascular Aneurysm Repair (EVAR) and to assess the relationship between these morphological features and the occurrence of endoleak and non-endoleak complications. Secondary endpoints included analyzing the rates and underlying causes of secondary interventions post-EVAR, as well as their effects on patient mortality and morbidity rates. Operational success was defined based on several criteria: the absence of conversion to open surgery, intraoperative mortality, Type 1 or Type 3 endoleaks, stent migration, and occlusion observed during complementary angiography.

### Computed tomographic angiography (CTA) measurements

Preoperative and postoperative morphovolumetric measurements of patients were conducted using the Sarus Workstation program. A cardiothoracic surgeon and a radiologist, each with 10 years of experience, performed a detailed comprehensive three-dimensional analysis. The volume of the aneurysm was quantified in cubic centimeters, and its density was assessed in Hounsfield Units (HU) across the proximal, distal, and middle regions. The average of these three measurements was calculated, and the data were transferred into an Excel spreadsheet for further analysis.

These measurements were derived from preoperative and postoperative thoracoabdominal computed tomographic angiography (CTA) scans. The CT examinations utilized 128 and 512-detector CT scanners (GE Healthcare). A total of 120 ml of contrast agent was administered at a rate of 4 ml/s, with images captured during the arterial phase (at the 20-s mark) using a slice thickness of 0.625 mm. Images were reviewed in axial, coronal, and sagittal planes, employing customized three-dimensional volumetric software (Advantage Workstation 4.2, GE Healthcare Technologies).

Total aortic and open lumen volumes were semi-automatically measured in cubic centimeters using this specialized software. The outer wall of the aneurysm sac was delineated circularly and measured using a semi-automatic method. The following parameters were used as reference points for volume measurements: Diameter A (infrarenal diameter), Diameter B (pre-sac diameter), Diameter C (aneurysm neck length), and the maximum diameter. Thrombus volume was calculated by subtracting the patent lumen volume from the total aortic volume. This methodology was consistently applied to both preoperative and postoperative CTA measurements.

## Statistical analysis

For clarity, *positive remodeling* was defined as a ≥10% reduction in aneurysm sac volume, which approximately corresponds to a ≥5 mm reduction in sac diamete**r** based on our regression model. Unless otherwise specified, references to “positive remodeling” in this manuscript denote volumetric changes.

All statistical analyses were conducted using the SPSS statistical software (SPSS for Windows 15.0, Inc., Chicago, IL, USA). Power analysis was performed with the statistical package program G*Power 3.1.9.7 (Franz Faul, Universitat Kiel, Germany); power = 98%. Continuous variables were tested for normal distribution using the Kolmogorov–Smirnov test. Continuous variables conforming to normal distribution were expressed as “mean ± standard deviation (SD),” and those not conforming to normal distribution were expressed as “median and interquartile range.” Categorical variables were presented as numbers and percentages. The Wilcoxon test was utilized for continuous variables. Patients were categorized into two groups based on the presence or absence of aortic positive remodeling. Demographic characteristics, perioperative variables, and follow-up parameters were compared using the “independent samples *t*-test” or “Mann–Whitney *U* test” for continuous variables and the “chi-square test” or “Fisher's exact test” for categorical variables. Independent predictors for postoperative aortic positive remodeling were determined using multiple regression analysis, and odds ratios with 95% confidence intervals were calculated. The regression analysis model included all variables with differences at the *p* < 0.1 level in univariate analysis.

The relationship between the reduction in sac volume during follow-up and the reduction in diameter was analyzed using linear regression. ROC analysis was employed to present sensitivity and specificity rates for the relationship between the aneurysm neck angle and Type 1A endoleak and sac volume reduction. Sensitivity and specificity rates for the relationship between postoperative CT findings and secondary intervention were presented through ROC analysis. A *p*-value < 0.05 was considered statistically significant. Survival analysis was conducted using Kaplan–Meier analysis for patients with positive remodeling and those without.

## Results

The operative success rate was 100%. Of the 131 patients monitored, 93.1% were male, with an average age of 69.08 ± 7.51 years. Hypertension (HT) was present in 71.8% of the patients, coronary artery disease (CAD) in 48.1%, and 53.4% had a history of smoking. Additionally, 5.3% of the patients had a history of cancer.

General anesthesia was administered to 84.5% of the patients. The mean fluoroscopy time was 10.4 ± 7.5 min, and the average contrast volume used was 55.5 ± 25.9 cm^3^. The average duration of stay in the intensive care unit was 7.1 ± 12.8 h, and the mean hospital stay was 2.9 ± 2.8 days. The mean follow-up period was 25.6 ± 15.02 months.

The preoperative mean aneurysm diameter was 65.5 ± 13.3 mm (range: 54 mm to 118 mm). Diameter A (infrarenal diameter) measured 23.4 ± 3.5 mm, Diameter B (pre-sac diameter) was 24.75 ± 4.4 mm, and the aneurysm neck length was 28.7 ± 13.1 mm. The average alpha angle was 39.04 ± 25.25°. The mean oversizing ratio of the proximal stent graft was 16.8 ± 5.2%. A mild but significant correlation was observed between oversizing and postoperative increase in infrarenal neck diameter (*r* = 0.42, *p* = 0.015), suggesting that greater oversizing may contribute to neck dilatation during follow-up.

The total aneurysm sac volume was 221.7 ± 146.4 cm^3^ (range: 44 cm^3^–873 cm^3^), and the thrombus volume averaged 104.6 ± 88.6 cm^3^ (range: 0–410 cm^3^). The mean preoperative ratio of thrombus volume to total aneurysm volume was 44.5% ± 22.3%. The thrombus density averaged 35.7 ± 13 Hounsfield Units (HU). Localization of the thrombus within the sac was circular in 49.6% of cases. The mean diameter of the inferior mesenteric artery (IMA) was 2.2 ± 1.1 cm. Additionally, 57% of patients had four or more patent lumbar arteries.

Type 2 endoleak was observed in 8.3% (11 patients), and intrasacral thrombus was located posteriorly in 7.6% (10 patients) of the cohort.

A total of 83.2% of the patients were receiving beta-blockers or other antihypertensive treatments. Statin use was observed in 42% of the cases, while metformin was used by 10.7% of the patients. Approximately 60.3% of the patients were on aspirin (ASA) monotherapy, and dual antiplatelet therapy was utilized by 32.8% of the patients.

During the follow-up period, endoleak was observed in 33 patients (26%). Specifically, 6 patients (5%) presented with Type 1A endoleak, 7 patients (5.3%) with Type 1B endoleak, 14 patients (10.7%) with Type 2 endoleak, and 6 patients (5%) with Type 3 endoleak.

There was no early mortality observed. Late-term mortality occurred in 21 patients (16%), with the most common cause being cardiac-related (38%). Aneurysm-related mortality was documented in 2 patients (1.5%) who had Type 3 endoleak and declined reinterventional treatment. Postoperatively, secondary interventions were performed in 17 patients (12.9%) due to endoleak, resulting in a total of 19 patients (14.5%) undergoing additional procedures. Iliac occlusion was identified in 2 patients (1.5%), one of whom underwent femorofemoral crossover bypass, while the other underwent femoral embolectomy with iliac extension.

Postoperative morphological changes in comparison to preoperative measurements are presented in Table 1. Although there was a significant reduction in aneurysm diameter postoperatively, no significant decrease in aneurysm volume was observed. Both Diameter A and Diameter B showed significant increases (*p* < 0.001), while the alpha angle significantly decreased postoperatively (*p* < 0.001).

**Table 1:** Change in preoperative and postoperative morphological measurements.

Parameter	Preoperative (mean ± sd)	Postoperative (mean ± sd)	*P* value
Aneurysm diameter (mm)	65 ± 13	62 ± 16	<0.001
Diameter A (infrarenal diameter mm)	23 ± 3	26 ± 4	<0.001
Diameter B (diameter before sac mm)	24 ± 4	24 ± 4	<0.001
Aneurysm neck Length(mm)	29 ± 13	30 ± 13	*P* = 0.021
Alpha angle (degrees)	39 ± 25	32 ± 23	<0.001
Aneurysm volume (cm^3^)	221 ± 146	215 ± 162	*P* = 0.487
Thrombus volume (cm^3^)	104 ± 88	159 ± 145	<0.001
Thrombus density (HU)	35 ± 13	34 ± 11	*P* = 0.177
Thrombus/aortic volume %	44 ± 22	65 ± 16	<0.001

SD, standard deviation.

**Figure 1:**
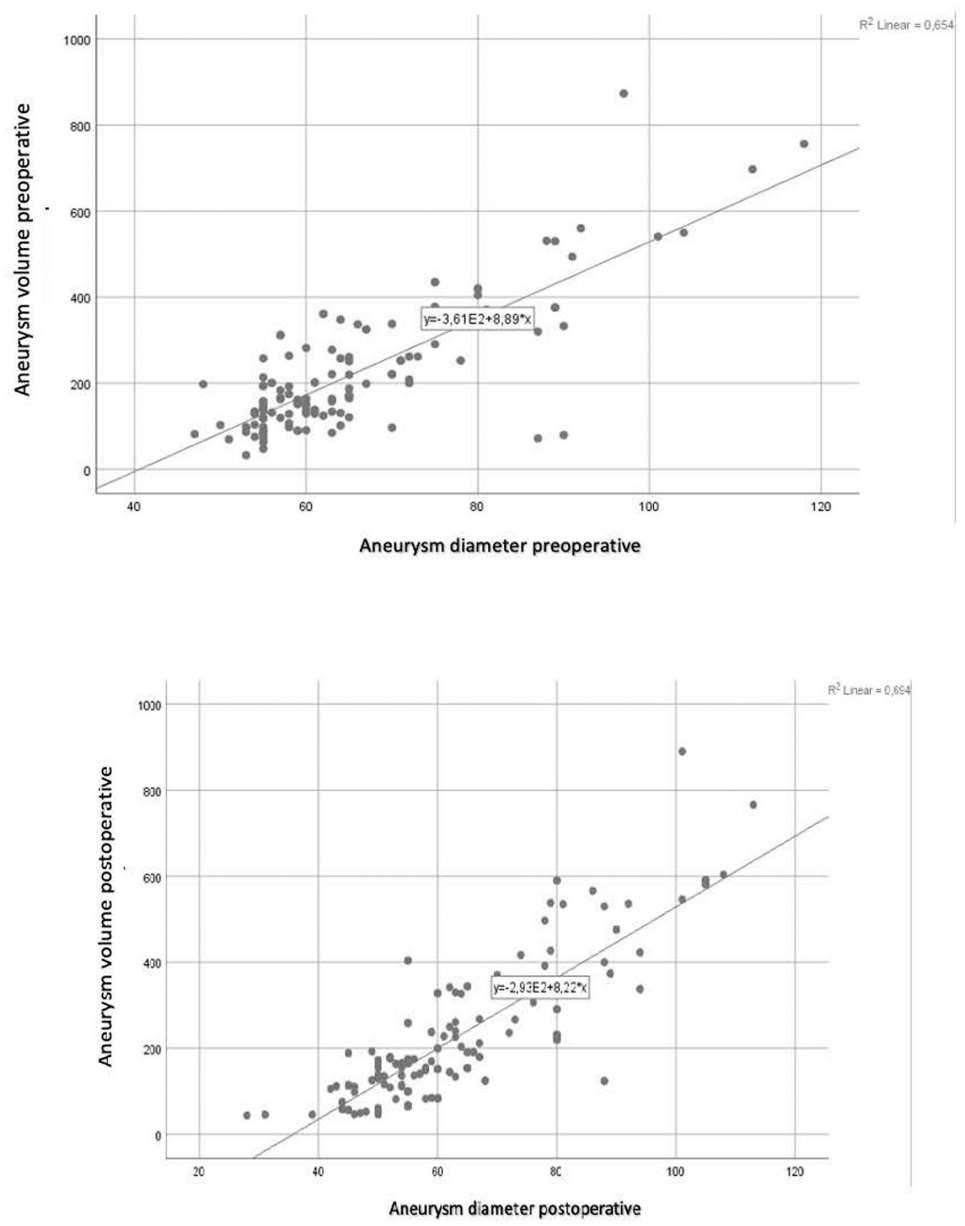
Preoperative and postoperative diameter-volume correlation.

A positive correlation was found between preoperative and postoperative aneurysm diameter and volume, with correlation coefficients of *r* = 0.809 and *r* = 0.833, respectively (Figure 1). The relationship between diameter and volume differences is articulated by the following equation:

Diameterdifference=4.464±0.085xVolumedifference%

According to this model, a 10% change in aneurysm sac volume corresponds to an approximate 5.31 mm change in diameter. A volume decrease of 10% or more was classified as positive remodeling. Based on this criterion, 44 patients (33.5%) exhibited positive remodeling during the follow-up period. A decrease in sac diameter was noted in 90 patients (69%), and a reduction in volume was observed in 69 patients (53%). Specifically, 50 patients (38.2%) experienced a decrease of 5 mm or more in aneurysm sac diameter, indicative of positive remodeling.

Comparative analysis of patients with positive remodeling vs. those without revealed significant differences in statin use (*p* = 0.04) and dual antiplatelet therapy (*p* = 0.035). Furthermore, the presence of thrombus in the neck (≥25%, *p* = 0.049), having ≤3 patent lumbar arteries (*p* < 0.001), inferior mesenteric artery (IMA) diameter (*p* = 0.071), and endoleak presence during follow-up (*p* = 0.003) were significant in univariate analysis (Table 2). Multivariate logistic regression analysis was conducted on parameters with a *p*-value below 0.10 (Table 3).

**Table 2:** Comparison of patients with and without positive remodeling.

Parameter	Non-positive remodeling				Positive remodeling (≥%10 volume decrease)				
	Mean	SD	Number (*n*)	Percent (%)	Mean	SD	Number (*n*)	Percent (%)	*P* value
AD (mm)	66.15	13.50			64.14	12.99			0.213
Diameter A(mm)	23.74	3.88			22.66	2.58			0.190
Diameter B(mm)	24.91	4.56			24.41	4.03			0.614
TN									
≦25%			71	81.6			29	65.9	0.049
>%25			16	18.4			15	34.1	
Preop. V cm^3^	220.39	151.06			224.48	138.53			0.821
Postop. V cm^3^	264.18	168.82			119.25	91.54			<0.001
NLA									
≤3			27	31.0			29	65.9	<0.001
≥4			60	69.0			15	34.1	
IMA D. (mm)	2.32	1.14			1.94	1.08			0.071
EC			29	33.3			3	6.8	0.001
SI			16	18.3			1	2.3	0.019

AD, aneurysm diameter; TN, thrombus in the neck; PREOP V, preoperative volume; POSTOP V, postoperative volume; NLA, number of lumbar arteries; IMA, inferior mesenteric artery; D, diameter; EC, endoleak is under control; SI, secondary intervention.

**Table 3:** Effect of significant parameters on positive remodeling.

Parameter	Univariate analysis				Multivariate analysis		
	OR	%95 CI	*P* value	OR		%95 CI	*P* value
Statin use	2.17	1.04–4.53	0.040	2.07		0.84–5.05	0.112
DAPT use	0.40	0.17–0.94	0.035	0.30		0.11–0.81	0.017
25% or more thrombus in the neck	2.30	1.01–5.24	0.049	2.13		0.81–5.56	0.123
3 or fewer lumbar arteries	4.30	1.99–9.29	<0.001	3.80		1.56–9.22	0.003
IMA diameter(mm)	0.74	0.54–1.03	0.071	1.03		0.70–1.53	0.886
Endoleak in control	6.83	1.95–23.95	0.003	6.16		1.62–23.41	0.008

The absence of dual antiplatelet therapy (DAPT) was associated with an odds ratio (OR) of 0.3 (95% CI: 0.11–0.81; *p* = 0.017), having ≤3 patent lumbar arteries with an OR of 3.80 (95% CI: 1.56–9.22; *p* = 0.003), and the absence of endoleak during follow-up with an OR of 6.16 (95% CI: 1.62–23.41; *p* = 0.008) as independent predictors for positive remodeling. Regarding the alpha angle, Receiver Operating Characteristic (ROC) analysis indicated that an alpha angle greater than 47.5° was associated with Type 1A endoleak, with a sensitivity of 75% and specificity of 69.7% (AUC = 0.670, *p* = 0.046).

Preoperative aneurysm volumes exceeding 233.5 cm^3^ were associated with secondary intervention, exhibiting a sensitivity of 80% and specificity of 76.7% (*p* = 0.001). Similarly, preoperative thrombus volumes exceeding 204 cm^3^ were linked to secondary intervention, with a sensitivity of 73.3% and specificity of 81.6% (*p* = 0.002).

**Figure 2:**
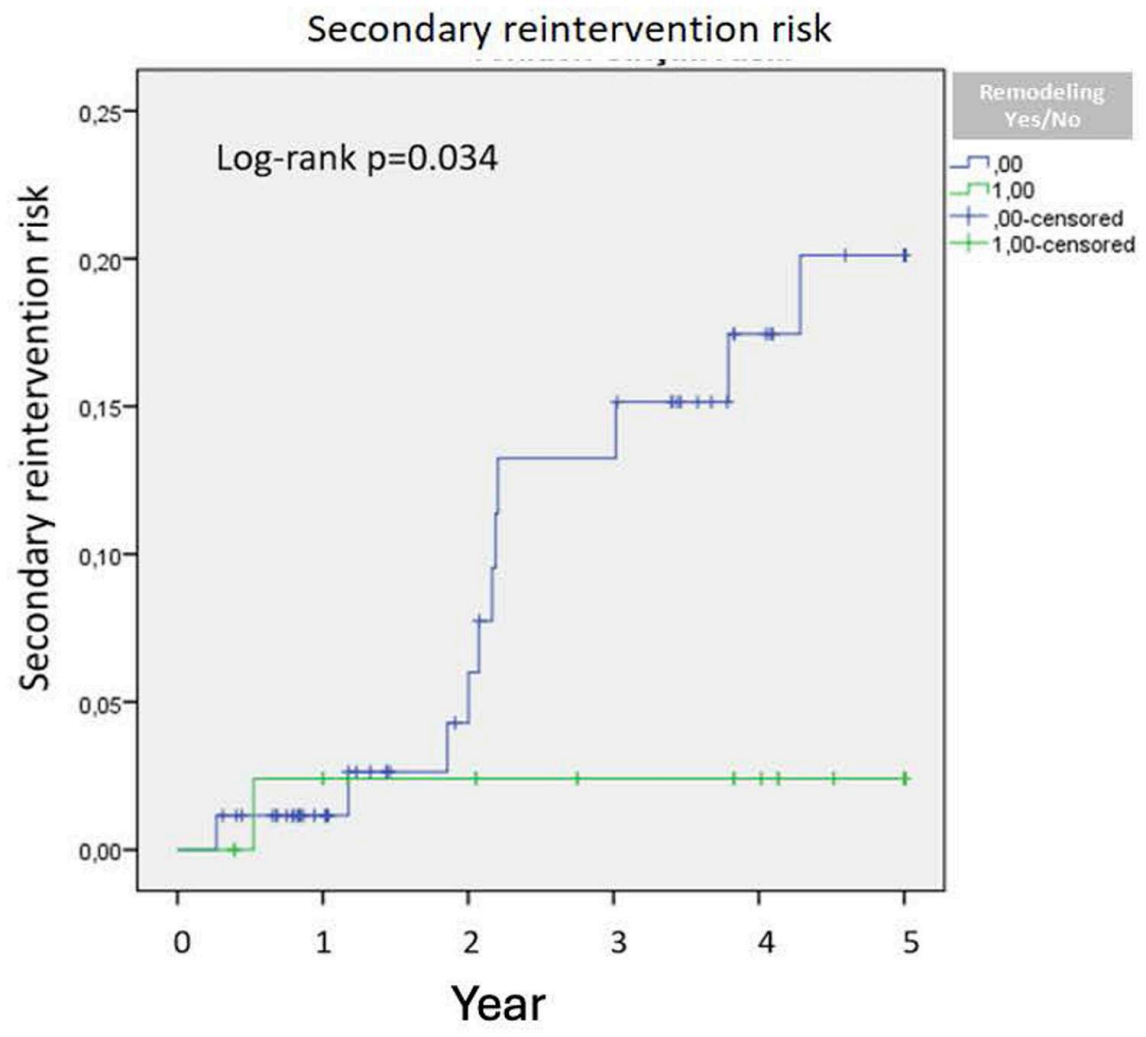
Secondary reintervention risk.

**Figure 3:**
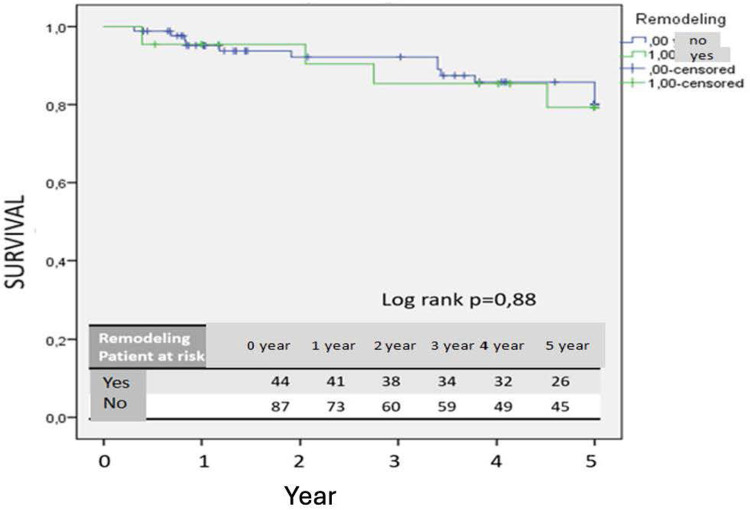
Kaplan–Meier survival curve.

Patients without remodeling after two years demonstrated a significantly increased risk of reintervention (*p* = 0.034) (Figure 2). No significant difference in 5-year survival was observed between patients with positive remodeling and those without (*p* = 0.88) (Figure 3).

Thrombus localization was categorized into four groups: no thrombus, anterior, posterior, and circular. Patients with posterior thrombus had significantly fewer patent lumbar arteries, averaging 3.1 ± 1.6, compared to other thrombus locations (*p* = 0.002). Notably, a decrease in aneurysm sac volume of 7% was exclusively observed in patients with posterior thrombus (*p* = 0.017). In contrast, patients with thrombus located in other regions exhibited an increase in aneurysm sac volume.

## Discussion

In the contemporary era, more than 70% of infrarenal abdominal aortic aneurysms are treated with endovascular aneurysm repair (EVAR) (1). Despite the minimally invasive nature of EVAR, it faces persistent criticism due to risks such as ongoing rupture, the need for re-intervention, continuous surveillance requirements, and associated costs. A critical issue remains surveillance noncompliance and the implementation of various surveillance algorithms. Recent studies have suggested that three-dimensional volumetric analysis of the aneurysm sac offers greater reliability than two-dimensional diameter measurements, particularly in the assessment of intrasac morphology (7). There is an ongoing debate regarding whether volumetric evaluation is more predictive than maximum diameter (Dmax).

In our study involving 131 patients, a positive correlation was identified between sac diameter and volume. The formulated relationship indicated that a 10% change in volume corresponds to approximately a 5.31 mm change in diameter. According to the Society for Vascular Surgery (SVS) reports, a 10% or more decrease in volume can be equated to a 5 mm diameter change and considered indicative of positive remodeling. Positive remodeling was observed in 44 patients (33.5%) during the follow-up period. The statistically higher incidence of endoleak and secondary interventions in patients without positive remodeling supports the utility of the 10% volume reduction criterion.

Lee et al. demonstrated that a 10% decrease in aneurysm sac volume post-EVAR is indicative of sac regression (10). Similarly, Franchin et al. reported that a decrease of 16% or more in aneurysm sac volume signifies sac shrinkage and noted that aneurysm-related mortality occurs in patients without volume reduction (8). Our study similarly underscores the efficacy of volumetric analysis, as the cohort without positive remodeling exhibited higher frequencies of endoleak and secondary interventions. An increased rate of secondary interventions was observed in patients without remodeling after two years, and no significant difference in five-year survival was noted between those with positive remodeling and those without. This may be attributable to prolonged follow-up, leading to an increased incidence of secondary interventions

Following endovascular aneurysm repair (EVAR), anatomical changes within the aneurysm sac are observed. In our study involving 131 patients, significant increases in Diameter A (infrarenal) and Diameter B (pre-sac) suggest the potential influences of oversizing and radial force post-EVAR. The observed postoperative increase in infrarenal and pre-sac diameters may partly reflect the radial force exerted by oversized stent grafts. Our correlation analysis demonstrated that higher oversizing ratios were associated with neck dilatation, consistent with previous reports highlighting the mechanical effect of graft radial expansion on adjacent aortic segments. This finding underlines the importance of optimized sizing to balance sealing efficacy and long-term neck stability. Concurrently, the significant reduction in infrarenal angulation (alpha angle) further highlights these anatomical changes. The observation that postoperative aneurysm diameter remained unchanged in some patients, alongside increases in volume and the incidence of endoleak with subsequent secondary interventions, underscores the critical need for volumetric analysis in postoperative surveillance.

Our findings indicate that sac volume measurement more accurately reflects changes in sac size that are not detectable through diameter changes alone. Further, the observed increase in volume in patients with minimal or no change in diameter underscores ongoing aneurysm sac dynamics, revealing that diameter measurements may be insufficient for surveillance purposes.

This study identifies several predictors of negative remodeling, including the preoperative detection of more than four patent lumbar arteries, the postoperative use of dual antiplatelet therapy (DAPT), and the presence of any type of endoleak. Additionally, it was observed that an aneurysm volume exceeding 233 cm^3^ and an intrasac thrombus volume greater than 204 cm^3^ were significantly associated with the need for secondary interventions.

The success of endovascular aneurysm repair (EVAR) is significantly influenced by the meticulous evaluation of preoperative anatomical features. Li et al. demonstrated a correlation between low thrombus volume and a high number of lumbar arteries with the occurrence of Type 2 endoleaks (11). In contrast, Yeung et al. found that patients with substantial thrombus volume exhibited lower rates of sac regression (12).

In our study, the presence of four or more lumbar arteries (*p* = 0.003) and the presence of any type of endoleak (*p* = 0.008) were associated with a lack of sac shrinkage. Another critical factor influencing both lumbar artery count and sac volume reduction is the localization of intrasac thrombus. Our findings indicate that the most significant sac shrinkage and the fewest lumbar arteries were observed in patients with posterior thrombus placement. Furthermore, the circular placement of thrombus, which includes posterior, anterior, and lateral walls, appears to impede sac shrinkage. This underscores the importance of thrombus anatomical localization in aneurysm remodeling.

Our study also established that a preoperative sac volume exceeding 233.5 cm^3^ and a thrombus volume greater than 204 cm^3^ are significant predictors of the need for secondary intervention. The elevated values of sac and thrombus volumes suggest that larger aneurysms are more susceptible to secondary interventions. This finding highlights the critical role of volumetric analysis in selecting the appropriate treatment modality for such patients. Montelione et al. further corroborated this by demonstrating that volumetric analysis is more effective than diameter measurements in determining the treatment approach (13).

**Figure 4:**
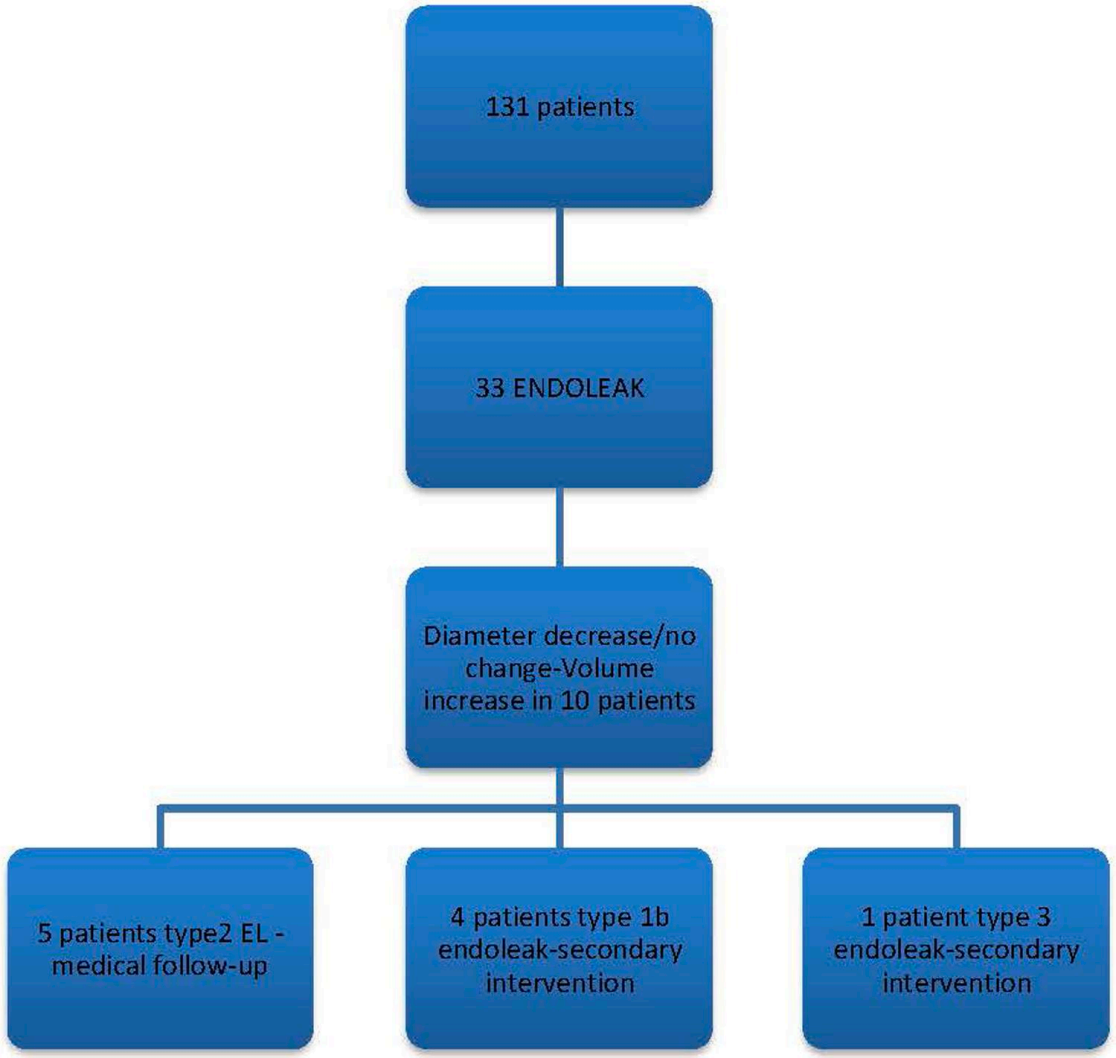
Follow-up findings in patients with a decrease or no change in diameter.

In our study, a decrease in sac diameter was observed in 90 patients (69%), and volume reduction occurred in 69 patients (53%). Specifically, 50 patients (38.2%) experienced a reduction in aneurysm sac diameter of 5 mm or more, indicating positive remodeling. According to volumetric analysis, positive remodeling was seen in 44 patients (33.5%). Notably, there were 10 patients with no change in diameter following EVAR, and in some instances, an increase in volume was observed despite a decrease in diameter. Among these patients, five exhibited Type 2 endoleak, four required secondary intervention due to Type 1b endoleak, and one underwent secondary intervention due to Type 3 endoleak (Figure 4).

These findings highlight a strong positive correlation between diameter and volume; however, in cases such as Type 2 endoleak, characterized by slow flow, diameter measurements may be misleading. Volume changes are observed earlier, thus necessitating the correlation of diameter measurements with volumetric analysis. Keulen et al. demonstrated that morphological changes not detectable by transverse and orthogonal diameter measurements can be identified through volume measurements, and conversely, diameter increases can occur without a concomitant volume increase (7).

Our results affirm the importance of correlating diameter with volumetric analysis, as sole reliance on either metric can lead to misinterpretation. Bargelli et al. indicated that the absence of a 0.3% volume decrease at a six-month follow-up strongly predicts endoleak (14). It has also been observed that aneurysms exhibiting no volume change shortly after EVAR are indicative of endoleak, particularly for slow-flow endoleaks such as Type 2 endoleak (15, 16).

Post-endovascular aneurysm repair (EVAR) remodeling is influenced by both anatomical factors and medical treatment. The use of antiplatelet therapy remains controversial; a recent study found that antiplatelet therapy was associated with no sac regression during the first year and subsequent late sac expansion (17). In our study, the absence of double antiplatelet therapy (aspirin and clopidogrel) was found to be three times more strongly associated with positive aneurysm remodeling (*p* = 0.017). Statins, which are known to reduce cardiovascular mortality, also have pleiotropic effects, including anti-inflammatory activity, reduction of protease activity involved in aneurysms, and improvement of endothelial function. Raux et al. demonstrated that post-EVAR statin use is effective in promoting sac regression (18). However, in our study, only 54% of the 44 patients with positive remodeling were on statins, which may indicate an inadequacy in assessing the full impact of statins on remodeling outcomes.

The methodology for performing volumetric analysis during post-EVAR follow-up typically relies on contrast-enhanced computed tomography (CT) measurements. Challenges such as age-related decline in estimated glomerular filtration rate (eGFR), risk of contrast-induced nephropathy, and patient allergies to contrast agents complicate the use of contrast-enhanced CT scans. In our clinic, Color Doppler Ultrasound (CDUS) is frequently utilized for post-EVAR follow-up. Our findings showed a strong correlation between contrast-enhanced CT angiography and CDUS, particularly in measuring the diameter of Type 1 and Type 3 endoleaks (19). Causey et al. suggested that expanding aneurysms could be identified earlier through volumetric analysis compared to diameter measurements during post-EVAR follow-up, and highlighted the safety and efficacy of 3D ultrasound for this purpose (20).

In our study, volumetric measurements were performed using a semi-automatic method on 3D customized workstations. The primary disadvantage of this method is its time-consuming nature and reliance on specific software. However, with the advancement of artificial intelligence technology, volumetric analysis can now be conducted more rapidly and safely. It is conceivable that, in the future, aneurysm sac volume could be measured almost simultaneously with diameter, thereby becoming a key parameter for secure monitoring (21).

Our study highlights the potential for late secondary interventions post-EVAR, reinforcing the necessity for lifelong monitoring. It also emphasizes that exclusive reliance on maximum diameter (Dmax) measurements during follow-up can be misleading. Effective and safe surveillance is achievable only through a combination of diameter and volume assessments, as demonstrated by the results of our study.

## Limitations

Several limitations of our study should be acknowledged. A substantial portion of the follow-up period overlapped with the COVID-19 pandemic, which impeded the regular performance of CT angiography measurements. The study was conducted independently of EVAR graft brand manufacturers, introducing a variable that could affect the results due to potential differences in radial strength among various grafts. Given the small sample size and the low incidence of adverse events, our study may not have identified specific or significant relationships.

## Conclusion

Our study found that the use of dual antiplatelet therapy (DAPT) and the presence of more than three patent lumbar arteries may inhibit sac shrinkage. Conversely, the presence of a posteriorly located thrombus could positively influence sac shrinkage by promoting the potential thrombosis of lumbar arteries over time.

Given the three-dimensional structure of the aneurysm sac, volumetric monitoring proves to be particularly valuable in patients with endoleaks or those exhibiting stable diametric measurements. The correlation between maximum sac diameter and volume underscores the necessity for cross-referencing these parameters. Nevertheless, further research with larger sample sizes is required to explore these aspects more comprehensively. This approach could lead to the development of the most appropriate and cost-effective surveillance method.

## Data Availability

The raw data supporting the conclusions of this article will be made available by the authors, without undue reservation.
